# Fintech Risk Management: A Research Challenge for Artificial Intelligence in Finance

**DOI:** 10.3389/frai.2018.00001

**Published:** 2018-11-27

**Authors:** Paolo Giudici

**Affiliations:** Department of Economics and Management, University of Pavia Pavia, Italy

**Keywords:** regulatory technology, supervisory technology, blockchain, big data analytics, artificial intelligence, peer-to peer lending (p2p lending), robo-advisory, cryptoassets

## Overview

The Financial Stability Board (2017b) defines FINancial TECHnology as “technologically enabled financial innovations that could result in new business models, applications, processes, or products with an associated material effect on financial markets and institutions and on the provision of financial services.”

While innovation in finance is not a new concept, the focus on technological innovations and its pace have increased significantly. Fintech solutions that make use of big data analytics, artificial intelligence and blockchain technologies are currently introduced at an unprecedented rate. These new technologies are changing the nature of the financial industry, creating many opportunities that offer a more inclusive access to financial services. The advantages notwithstanding, FinTech solutions leave the door open to many risks, that may hamper consumer protection and financial stability. Relevant examples of such risks are underestimation of creditworthiness, market risk uncompliance, fraud detection, and cyber-attacks. Indeed fintech risk management represent a central point of interest for regulatory authorities, and require research and development of novel measurements.

Across the world, there is a strong need to improve the competitiveness of the fintech sector, introducing a risk management framework that can supervise fintech innovations without stifling their economic potential. A framework that can help both fintechs and supervisors: on one hand, fintech firms need advice on how to identify opportunities for innovation procurements, for example in advanced regulatory technology (RegTech) solutions; on the other hand, the supervisory bodies' ability to monitor innovative financial products proposed by fintechs is limited, and advanced supervisory technology (SupTech) solutions are required. A crucial step in transforming compliance and supervision is to develop uniform and technology-driven risk management tools which could reduce the barriers between fintechs and supervisors.

We believe that a focused international research activity, coordinated at the level of a highly reputed open access scientific journal with multiple key foci, such as **Frontiers in Artificial Intelligence**, can help to close the gap between technical and regulatory expertise, in particular providing risk management procedures common to both sides. It could lead to the development of a regulatory framework that encourages innovations in big data analytics, artificial intelligence and blockchain technologies which, at the same time, satisfies supervisory concerns to apply regulations in an effective and efficient way and which protect consumers and investors.

Regulations and related supervisory requirements are placing great focus on risk management practices, which in turn drives the need for deep, transparent and auditable data analyses across organizations. Technologies such as big data analytics, artificial intelligence and blockchain ledgers may address risk management requirements and the associated costs more efficiently. In particular, these technologies can: (i) reduce credit scoring bias and improve fraud detection in peer-to-peer lending; (ii) measure and monitor systemic risk in peer-to-peer lending; (iii) measure and monitor market risk and volatility in financial markets (iv) enhance client risk profile matching in robo-advisory; (v) identify illegal activities in crypto markets, including fraudulent initial coin offerings and money laundering; (vi) identify and prioritize IT operational risks and cyber risks.

In line with these developments, the specialty section “Artificial Intelligence in Finance” of *Frontiers in Artificial Intelligence* aims to create an international research forum that provides and publishes key research on shared risk management solutions that automatize compliance of fintech companies (RegTech) and, at the same time, increases the efficiency of supervisory activities (SupTech). The Artificial Intelligence in Finance section also builds synergies with the broader tech-focused specializations with its own journal and within *Frontiers in Big Data* and *Frontiers in Blockchain*.

Currently, supervisors and fintechs do not have a common framework to understand the opportunities/risks balance, leading to different perceptions. **Artificial Intelligence in Finance** aims to provide a research forum to discuss solutions that efficiently automatize both fintech compliance (RegTech) and supervisory monitoring (SupTech).

The vision of **Artificial Intelligence in Finance** is to build a collaborative innovative environment from which both supervisory bodies and regulated institutions can benefit. Specifically, we aim at connecting the two sides of the coin by organizing a forum for research discussion which will have the purpose of sharing risk measurement solutions that fit the needs of both regulated institutions and regulators. The discussion will draw on the contribution from three types of project participants:

- Fintech and financial companies, who have a detailed understanding of business models based on financial technologies;
- Regulators and supervisors, who have a detailed understanding of the regulations and risks that concern financial technologies;
- Universities and research centers, which have a detailed understanding of the risk management models that can be applied to financial technologies.

Conceptually, the research content of the journal will be classified around three types of FinTech risk management models, which will constitute the conceptual map of the journal The classification is based on the three main technologies that drive FinTech innovations:

- **Big data analytics**, with its application to peer-to-peer lending, with main risks arising from credit risk, and systemic risk;
- **Artificial intelligence**, with its application to financial robo-advice, with main risks arising from market risk and compliance risk;
- **Blockchain**, with main application to crypto-assets, with main risks arising from fraud detection, money laundering risk, IT operational risk and cyber risks.

Artificial Intelligence in Finance will consider research from all the above three areas. Research in Big data and Blockchain, but also in AI more generally, neatly connects to other Frontiers' journals, such as Frontiers in Big Data and Frontiers in Blockchain. This interdisciplinary infrastructure aims to leverage collaborations and the expertise of diverse research communities—something that is at the center of FinTech innovation.

### Innovative technologies

The European Commission (2018) argues that the term big data refers to “*large amounts of different types of data produced with high velocity from a high number of various types of sources.”* Big data analytics refers to the variety of technologies, models and procedures that involve the analysis of big data aimed revealing insights, patterns of causality and of correlation, and to predict future events (similarly to data science and to its predecessor, data mining: see e.g., Giudici, 2003).

Over the years, academics and experts in computer science and statistics have developed advanced techniques to obtain insights from large datasets combining a variety of data types obtained from a variety of sources (see Brito, 2014). These models are able to utilize the ability of computers to perform complicated tasks by learning from experience. Following a definition offered by the Financial Stability Board (2017a) artificial intelligence is a broad term capturing “*the application of computational tools to address tasks traditionally requiring human sophistication*.” It is important to mention that often the terms AI and machine learning are used interchangeably. However, Artificial Intelligence is a broader term, of which machine learning represents a subcategory: the difference being that machine learning is a data-driven way to achieve AI, but not the only one; similarly, big data analytics is broader then machine learning, as it includes also statistical learning. For a further discussion on the difference between AI and Machine Learning, see also Kersting (2018).

Among the emerging technologies with significant potential to change the financial systems and industry from its core, the blockchain has received a significant amount of attention over the last few years. A blockchain is a distributed database of records of all transactions or digital events that have been executed and shared among participating parties (De Filippi and Hassan, 2016). Each transaction in the distributed database of records is verified by the participants through a majority consensus and, once confirmed, the transaction can never be altered or deleted (see for e.g., Tasca and Hayes, 2016). Hence, the blockchain contains a certain and verifiable record of every single transaction ever made between the participants in a network (see e.g., Pontiveros et al., 2018).

### Financial applications

Many fintech applications rely on big data analytics and, in particular, those based on peer-to-peer (P2P) financial transactions, such as peer to peer lending, crowdfunding, and invoice trading. The concept *peer-to-peer* captures the interaction between units, which eliminates the need for a central intermediary. In particular, peer-to-peer lending enacts disintermediation by allowing borrowers and lenders to communicate directly, using the platform as an information provider which, among other things, assesses the credit risk of borrowers. From a regulatory perspective, a key point of interest is whether such credit risk measurements reflect the actual capacity of borrowers to repay their debt. Regulation must be technologically neutral and, therefore, credit risk compliance should be imposed on fintechs as they are for banks. At the same time, it cannot be so burdensome to disincentivise the growth of alternative financial service provides (see Talonen et al., 2016).

Automated consultants, known as robot advisors, are considered the main application of AI in financial services. The European Supervisory Authorities joint report defines the phenomenon of automation in financial advice as “*a procedure in which advice is provided to consumers without, or with very little human intervention and with providers relying on computer-based algorithm and/or decision trees*.” In practice, robot advisors build personalized portfolios for investors, on the basis of algorithms that take into account investors' information such as age, risk tolerance and aversion, net income, family status. Obtaining this information is a legal requirement and robot advisors employ online questionnaires to obtain it.

Crypto assets are the main application of blockchain technology and are considered one of the largest markets in the world which remain unregulated. Within the last decade, digital currencies, operating independently of central banks have massively grown in popularity, price, and volatility. The Bitcoin is the oldest, most popular and widely used digital currency, and it offers low-cost, decentralized transfer of value anywhere in the world with the only constraint representing the availability of an internet connection. However, many other crypto assets are available, and new ones are continuously emerging through Initial Coin Offerings, in which a company sells digital tokens that eventually can be exchanged for goods, services or other currencies. It is a new fundraising method, which combines elements of both crowdfunding and traditional initial public offerings.

### Risk concerns and management

Although there are many existing legislations that are intended to serve in the interest of consumer and investor protection, lending fintechs give rise to “disintermediation,” which requires the need for further protection of consumers and investors. In the case of peer to peer lending, there are two main causes of concern. First, P2P platforms have less information on their borrowers, compared to classical banks, and are less able to deal with asymmetric information. Second, in most P2P lending platforms the credit risk is not held by the platform but, rather, by the investors. Both causes lead to a high likelihood that the scoring system of P2P lenders may not adequately reflect the “correct” probability of default of a loan. A further issue associated with the nature of P2P platforms is that they give rise by construction to globally interconnected networks of transactions. This suggests that they cannot avoid the measurement of systemic risks arising from contagion mechanisms between borrowers.

In the context of P2P lending, a key risk to measure is the risk associated with the default of borrowers: credit risk. Statistical theory offers a great variety of supervised models for credit scoring and credit risk management and, in particular, logistic regression, and generalized linear models (Bernè et al., 2006). The same models can be applied to similar classification problems in peer-to-peer lending, such as consumer's fraud and money laundering detection.

A key issue that arises in employing generalized linear models for P2P classification problems is that the event to be predicted is multivariate. To solve this issue, Lauritzen (1996) introduced graphical models to model dependencies between random variables, by means of a unifying and powerful concept of a mapping between probabilistic conditional independences, missing edges in a graphical representation, and suitable statistical model parametrisations. In parallel, Mantegna (1999) introduced hierarchical structures in financial markets, based on correlation matrices, developing a powerful distance-based statistical model able to uncover similarity relationships among financial assets. These models have been applied in a variety of financial contexts, including credit scoring, churn modeling, and fraud detection (for a review see Giudici, 2003; Guegan and Hassani, 2017).

In line with these developments, Giudici and Hadji-Misheva (2018) suggest to model credit risk of peer to peer lending taking advantage of their natural interconnectedness, by means of correlation network models, a subset of graphical models that has been introduced in finance to measure systemic risks risk (see e.g., Arakelian and Dellaportas, 2012; Battiston et al., 2012; Billio et al., 2012; Diebold and Yilmaz, 2014; Výrost et al., 2015). This allows to improve the accuracy of credit risk models and, furthermore, to measure a risk type that is particularly evident in P2P lending: systemic risk, recently applied to bank, and sovereign default. Giudici and Hadji-Misheva (2018) show how to build a correlation network for P2P lending: associating each borrower with a statistical unit, at each time point many variables can be observed for that unit; in the case of SME lending, balance sheet variables; in the case of consumer credit, transaction account variables. A correlation network (Mantegna, 1999) between borrowers can then be built on the basis of the observed values of one variable over time. Associating each borrower with a node in the network, each pair of nodes can be thought to be connected by an edge, whose weight is equal to the correlation coefficient between the two-time series of the chosen variable, each corresponding to a specific borrower. If we consider all pairs of borrowers, we will get a matrix of correlation weights, also known as “adjacency matrix.” Once the adjacency matrix is derived, summary network centrality measures suggest which are the most important units in the network or, in financial risk terms, which are the most contagious borrowers (Giudici and Spelta, 2016; Tomašev et al., 2016). Furthermore, Giudici and Hadji-Misheva (2018) show, in real P2P lending data analysis that, when network centrality measures are embedded in a generalized linear model specification, they can improve the predictive accuracy of credit scoring algorithms.

Moving to asset management fintechs, note that the advantages associated with automatized advice may be offset by the greater risks that are brought on board, among which the risks of making unsuitable decision (due to lack of information or reduced opportunities) and risks of errors and functional limitations of the tool. As is the case with big data analytics, there are several regulatory requirements that already exist and apply to automated advice. However, some risks are yet to be fully considered and measured. Among them, we believe the following are the most relevant: (i) compliance risk—mismatch between expected and actual investment risk class; (ii) market risk—the likelihood that adverse movements and volatility in financial markets, either traditional or new (crypto markets) cause unexpected losses in investors' portfolios.

As for peer to peer lending, the increased risks connected with the use of robot advisory platforms can be mitigated by an appropriate analysis of the data they generate. In this respect, robot advisors generate, in an automated way, a large amount of data, which can be leveraged not only to improve the service, making it more personalized, but also to reduce compliance risk and, in particular, the risk of an incorrect profile matching between “expected” and “actual” risk classes (see e.g., Valkanov, 2016).

Recent studies have shown that an accurate analysis of risk propensity questionnaires can allow robo-advisors to estimate the “expected” risk class of each investor. Data analysis algorithms can be implemented also on the supply side, considering the returns of the available financial products to classify them into homogeneous “actual” risk classes. Linking together the “expected” risk classification of an investor with its “actual” classification allows to evaluate whether a robot advisor respects its risk profile (Kabašinskas et al., 2017), one of the most important requirement of the MIFID regulation, which thus becomes, in the context of robot advisory, a verifiable requirement, not only from a formal viewpoint, but also from an operational one.

The literature on the measurement of expected risks in robot advisory is very limited. Scherer (2016) investigates, within a machine learning approach based on tree models, the key investor characteristics that can predict financial market participation; Alexy et al. (2016) is a related work. Similarly, the literature on the measurement of the actual risk of a given set of financial products is also very limited. Tumminello et al. (2005) and Tola et al. (2008), who employ clustering models to construct homogeneous asset classes, and (Baitinger and Papenbrock, 2017), who considered interconnectedness risk, are noticeable exceptions.

Giudici and Polinesi (2018) extend Scherer's approach deriving expected risk classes from the responses to the MIFID questionnaire, building correspondence analysis models on the observed contingency table, that results from the cross-classification of the responses to the questionnaire. They also show how to employ feed forward neural network models to estimate the risk class of a given investor's portfolio, on the basis of the observed returns. By comparing the expected with the actual risk class, for a sample of investors, it is thus possible to evaluate, in an automated way, whether the robot advisor is compliant with the risk profile of the investor.

We remark that specific concerns arise, from a market risk viewpoint, when crypto assets are combined with classical ones in investment activities. In particular, bitcoins, and crytpoassets have been associated with exceptionally high volatility and greatly sensitive prices (see Jabłecki et al., 2015; Traian et al., 2017; Žiković Saša, 2017; Chen et al., 2018). Indeed, fluctuations are very common throughout the existence of the crypto assets, which in turn raises the question whether this behavior is attributed to general market conditions or to idiosyncratic factors (as discussed by Makrichoriti and Moratis, 2016). To address these concerns, network models take the central stage, as could be expected. Nakamoto (2009) described the bitcoin as a purely peer-to-peer version of electronic cash that allows online payments to be send directly from one party to another, without going through a financial institution. Hence, in its essence, the bitcoin represents a solution to the double-spending problem using a peer-to-peer network. This suggests that a correct measure of the risks associated with this technology must take into account the interconnections generated by network transactions.

In this context, correlation network models can be employed to detect the main determinants of volatility (as in Papenbrock and Schwendner, 2015; Barucci and Marazzina, 2016). More recently, (Giudici and Abu-Hashish, 2018) have applied correlation VAR models to check whether price contagion between different bitcoin exchange markets exist, and found that this is the case, especially for smaller exchanges.

Many innovative fintechs have payment deals with the application of blockchain technology. The main risk concerns about blockchain applications in finance relate to operational risks. Many international regulatory authorities have raised significant concerns suggesting that, in most cases, small investors do not adequately understand the risk involved with Initial Coin Offerings. Although many legitimate start-ups use ICOs for the purpose of raising money, many projects also exist which do not intend to deliver any value to the investors. The market has seen many such cases of fraudulent ICOs which raises deep concerns for investor protection and overall financial stability. To identify the main determinants of fraudulent ICOs, text mining analytics methods, that use network models to reduce their curse of dimensionality, can be applied. Following most recent statistics, 99% of all ICOs use Telegram as a channel for interacting with their communities. Typically, the Telegram groups are characterized by many members and detailed discussions about the value of the individual projects, as well as, by the expectations of the communities concerning the success of the ICO and the company. By collecting data from the Telegram ICOs (including the corresponding white papers) and discussions on Telegram chats relating the value and prospects of the projects in question, we can build, train, and test supervised models to discriminate and classify ICOs by their probability of fraud, using for example the methods shown in Hochreiter (2015).

Another cause of concern is that crypto assets allow for a multi-billion dollar global market of anonymous transactions, which does not undergo any control. Hence, its emergence and growth can create considerable challenges for market integrity, particularly from money laundering activities. Money laundering embraces all those operations to disguise the illicit origin of capital, to give it a semblance of legitimacy, and facilitate the subsequent reinvestment in the lawful economy. A recent study conducted by Foley et al. (2018) aims at quantifying and characterizing the illegal trade facilitated by the Bitcoin, to provide a better understanding of the nature and scale of the problem facing this technology. The results from the study suggest that approximately one-quarter of Bitcoin users and one-half of Bitcoin transactions are associated with illegal activity. The authors found that around $72 billion of illegal activity per year involves Bitcoin, which is close to the scale of the US and European markets for illegal drugs. In the context of money laundering detection, network-based community detection models can be employed. They exploit the transactional network topology for the purpose of identifying communities of users and, in particular, to identify communities of money launderers, using the transactions between them. More formally, the method that can be applied is a network cluster analysis algorithm that takes as inputs the set of users (“nodes” in network terminology) and the trades between users (“edges” or “links” in network terminology) (Foley et al., 2018). The output of the algorithm is an assignment of users to communities such that the “modularity” of the communities (density of links within communities and sparsity of links between communities) is maximized (Foley et al., 2018).

An additional cause of concern is that cryptoassets are fully digital and, therefore, may lead to higher IT operational risks, such as errors in the functioning of the algorithms, and hacking and manipulation of the algorithms (cyber attacks), to name only a few. While the literature on the quantitative measurement of operational risk constitute a reasonably large body (see for example Cruz, 2002), that on cyber risk measurement is very limited. As cyber risks are very different in nature, rare, and typically not repeatable, a useful approach to measure them is to consider an ordinal-based, scorecard approach, similar to that done in self-assessment-based operational risk management (see e.g., Giudici, 2015), in reputation measurement (see e.g., Cerchiello and Giudici, 2015) or in portfolio analysis using stochastic dominance (Post and Potì, 2016).

In this way a cyber risk measure can be used to rank cyber risks and prioritize interventions, preventing failures and reducing ex-ante the impact of risks. This on the basis of ordinal random variables, that represent the levels of frequency and severity for different cyber risk events, in different business areas. A similar approach can be consistently undertaken to measure operational risks deriving from the use of robo-advisors, caused by their malfunctioning, rather than by cyber-attacks. Note also that an ordinal-based measurement of operational risks and cyber risks can be easily adapted to scenario testing, which is one of the best ways for the financial industry to protect from them, specifically when they are conducted across the industry.

## Conclusions

In this paper, we have focused on the emerging topic of financial technology. We have first identified the main technological drivers of change: big data analytics, artificial intelligence, and blockchain technology; and their main financial applications: in banking (peer to peer lending); in asset management (robot advisory); and in payment systems (crypto assets).

Our vision is to encourage the development and the growth of financial technologies, making them sustainable, minimizing their possible negative impacts on consumers and investors. This goal can be achieved through the development of appropriate risk management methods, whose compliance burden can be limited by the technology itself.

To achieve this aim, the paper has presented the main risk concerns that arise with the development of the most important financial technologies, and has suggested research directions in risk measurement models, appropriate to manage and mitigate the involved risks.

A strict collaboration and open discussion between academics, fintech experts, and regulators can help move us ahead in this direction, developing fintech risk management models that, while limiting the negative impact of disrupting technologies, encourage their development. The journal *Frontiers in Artificial Intelligence*, with the research specialty Artificial Intelligence in Finance, will be key in fostering collaborations and stimulating research debates on risk management practices. The goal is to share with the community the best practices to measure fintech risks. Practices that could be employed to offer “automated” risk management tools, for both RegTech and SupTech purposes, thus making fintech innovations competitive and sustainable.

## Author contributions

The author confirms being the sole contributor of this work and has approved it for publication.

### Conflict of interest statement

The author declares that the research was conducted in the absence of any commercial or financial relationships that could be construed as a potential conflict of interest.
